# Feasibility of Mini-Clinical Evaluation Exercise as a Workplace-Based Assessment Tool for Respiratory Medicine Interns: A Prospective Interventional Study

**DOI:** 10.7759/cureus.104689

**Published:** 2026-03-04

**Authors:** Parikh K Hymn, Minal Patel, Zainab Laxmidhar

**Affiliations:** 1 Department of Respiratory Medicine, Shantabaa Medical College & General Hospital, Amreli, IND; 2 Department of Physiology, Pramukhswami Medical College - Bhaikaka University, Anand, IND

**Keywords:** foramative assessment, medical education, medical interns, mini-cex, respiratory diseases, workplace based assessment method

## Abstract

Background

India bears a high burden of chronic respiratory diseases, making it imperative for medical interns to acquire strong clinical reasoning, examination, and diagnostic skills in respiratory medicine. Traditional assessment methods often fail to evaluate real-time clinical performance and provide structured feedback. Workplace-based assessment tools such as the mini-clinical evaluation exercise (Mini-CEX), recommended under Competency-Based Medical Education (CBME), offer an opportunity to assess clinical competence through direct observation. However, evidence regarding its focused application in respiratory medicine during internship is limited.

Aim

To evaluate the feasibility and effectiveness of the Mini-CEX as a workplace-based assessment tool for assessing clinical competence in common respiratory diseases among medical interns.

Methodology

A prospective educational interventional study was conducted among 20 medical interns posted in the Department of Respiratory Medicine at a tertiary care hospital. Each intern underwent two Mini-CEX encounters during a one-week posting. Clinical performance was assessed across five domains using the standard Mini-CEX tool. Intern and faculty perceptions were recorded using validated 5-point Likert scale feedback forms. Knowledge gain was assessed using pre- and post-tests. Statistical analysis included descriptive statistics and paired t-tests. Internal consistency of the tool was assessed using Cronbach’s alpha.

Results

There was a statistically significant improvement across five Mini-CEX domains between Encounter-1 and Encounter-2 (p < 0.001). Mean overall competence scores increased from 28.50 ± 3.1 to 35.65 ± 2.5. Knowledge scores showed a significant improvement with a mean gain of 7.15 points (p < 0.0001; Cohen’s d ≈ 3.71). The mean time required per Mini-CEX session was 12.6 ± 3.14 minutes, with a 100% completion rate and no reported practical difficulties. Interns and faculty reported high levels of satisfaction and acceptability. The assessment tool demonstrated excellent reliability (Cronbach’s α = 0.91).

Conclusion

Mini-CEX is a feasible, reliable, and effective formative assessment tool for evaluating and improving clinical competence in respiratory medicine among medical interns. Its integration into internship training aligns well with CBME principles and enhances both clinical performance and knowledge acquisition through structured observation and feedback.

## Introduction

According to the Global Burden of Diseases Report 2017, India bears one of the highest burdens of chronic respiratory diseases, contributing to approximately 15.69% of the global burden of conditions such as chronic obstructive pulmonary disease (COPD), asthma, and tuberculosis [1]. India has the highest number of COPD cases worldwide, estimated at 55.23 million, and accounts for nearly 43% of global asthma-related deaths. Given this substantial disease burden, it is imperative that medical interns develop robust clinical reasoning, examination skills, and diagnostic competence in respiratory medicine.

Traditional methods of assessing interns, including long case presentations, written examinations, and subjective faculty impressions, are often episodic and lack structured, real-time feedback. To address this gap, competency-based medical education has been widely advocated to align assessment with real-world clinical performance [2].

The Mini-CEX is a Workplace-based assessment tool (WPBA) developed by the American Board of Internal Medicine to evaluate clinical skills through direct observation in real clinical settings. It is widely used in undergraduate and postgraduate medical education to assess competencies, including history taking, physical examination, clinical judgment, and communication skills [3]. Prior research has demonstrated that Mini-CEX functions primarily as a formative assessment tool, providing structured feedback that enhances learning and improves patient care [4]. Additionally, Mini-CEX has been shown to possess high fidelity and acceptability among both learners and assessors [5].

While several studies have examined the use of Mini-CEX as a WPBA tool, there is a relative scarcity of research focusing on its structured implementation and evaluation within respiratory medicine during internship, a critical transitional phase in clinical training. Furthermore, most existing studies have assessed Mini-CEX either across multiple specialties or over extended durations. The present study uniquely evaluates Mini-CEX during a short respiratory medicine posting, integrating performance assessment, knowledge gain (pre-post testing), feasibility, and learner and faculty perceptions, thereby providing a comprehensive evaluation of its educational impact.

## Materials and methods

Study design

This was a single-center, prospective educational interventional study conducted in the Department of Respiratory Medicine at a medical college in Western India. Objectives were to assess the feasibility of implementing Mini-CEX as a workplace-based assessment tool for evaluating clinical skills related to common respiratory diseases among medical interns. To identify strengths and weaknesses in interns' performance across domains such as history-taking, physical examination, clinical judgment, and communication. It also assess perception of faculty and interns for Mini-CEX in assessing clinical competence for respiratory diseases and the effectiveness of implementing mini-CEX through Pre and post-test scores. The study was carried out for 5 months from August 2025 to December 2025.

Study participants

The study included 20 medical interns posted in the Department of Respiratory Medicine during the study period who provided written informed consent. Interns were eligible for inclusion if they were actively participating in clinical patient care and had attended the Mini-CEX orientation session. Interns were excluded if they were on leave during the posting period, were not involved in direct patient care, or had not undergone Mini-CEX orientation. Three faculty members from the same department, trained in workplace-based assessment, participated as assessors.

As this was a prospective educational innovation study, a convenience sample of all interns rotating through the department during the study period was included. Similar Mini-CEX studies in medical education have used comparable sample sizes to evaluate feasibility and educational impact.

Study procedure

All the interns and 3 faculties have attended one hour orientation lecture about Mini-CEX. Each student underwent two Mini-CEX encounters during starting of their rotation in the Respiratory Medicine department and at the end of their rotation. It was conducted in Out Patient Department. Common respiratory disease cases were given to them.

Mini-CEX assessment tool

The Mini-CEX is a workplace-based assessment tool developed by the American Board of Internal Medicine to evaluate clinical skills in real-time settings. It was designed to evaluate seven core clinical skills, namely medical interviewing, physical examination, clinical judgment, professionalism/humanistic qualities, counseling, organization, and overall clinical competence, which were assessed using a nine-point Likert scale. Scores from 1 to 3 were categorized as unsatisfactory, scores from 4 to 6 as satisfactory, and scores from 7 to 9 as superior. Out of seven clinical skills we have assessed, five skills, namely medical interviewing, physical examination, clinical judgment, professionalism/humanistic qualities and counselling skills.

Data collection

Mini-CEX performance score was documented using a standardized Mini-CEX pro forma after each session. Time duration was recorded for both observation and feedback. After the end of each session, faculty and interns were provided feedback questionnaires. The feedback questionnaires were validated by two faculty experts and three faculty members from the medical education unit. Feedback form includes questionnaires based on a Likert scale in which 1 is strongly disagree, and 5 is strongly agree. It has open-ended questions on likes, dislikes and suggestions to improve (Appendices)

Statistical analysis

All data collected were analyzed through SPSS (version 26). Mini-CEX domain scores (e.g., history taking, physical exam, clinical reasoning, professionalism, communication) were summarized using Mean, median, standard deviation, minimum, and maximum scores. Scores will be interpreted using the standard Mini-CEX scale (1-9): 1-3 = Below Expectations,4-6 = Meets Expectations7-9 = Exceeds Expectations. Continuous data are presented as mean ± SD. Comparison between Encounter-1 and Encounter-2 (paired data) was done using a paired t-test (n = 20). A two-tailed p < 0.05 was considered statistically significant. Feedback from interns and faculty on the Mini-CEX tool was recorded on a 5-point Likert scale (1 = strongly disagree to 5 = strongly agree). Internal consistency of the instrument: Cronbach’s α = 0.91.

## Results

A total of 20 medical interns completed two Mini-CEX encounters each, resulting in 40 observed clinical encounters with a 100% completion rate. Domain-wise analysis demonstrated a statistically significant improvement across all five Mini-CEX domains between Encounter-1 and Encounter-2. Mean scores for history taking improved from 5.80 ± 0.83 to 7.15 ± 0.59, physical examination from 5.35 ± 1.04 to 6.95 ± 0.76, and clinical judgment/diagnosis from 5.35 ± 0.93 to 7.05 ± 0.76. Communication skills increased from 5.85 ± 1.39 to 7.15 ± 0.88, while professionalism scores improved from 6.15 ± 1.14 to 7.35 ± 0.88. All improvements were statistically significant (p < 0.001). Overall competence scores showed a marked rise from 28.50 ± 3.1 to 35.65 ± 2.5, reflecting a mean gain of 7.15 points. Domain-wise Mini-CEX scores for Encounter-1 and Encounter-2 are summarized in Table 1, demonstrating significant improvement across all assessed domains.

**Table 1:** Domain-wise Mini-CEX scores (Encounter-1 vs Encounter-2; n = 20)

Domain	Encounter-1 Mean ± SD	Encounter-2 Mean ± SD	Mean Difference (E2 − E1)	p value (two-tailed)
History taking	5.80 ± 0.83	7.15 ± 0.59	+1.35	p < 0.001
Physical examination	5.35 ± 1.04	6.95 ± 0.76	+1.60	p < 0.001
Clinical judgment / Diagnosis	5.35 ± 0.93	7.05 ± 0.76	+1.70	p < 0.001
Communication skills	5.85 ± 1.39	7.15 ± 0.88	+1.30	p < 0.001
Professionalism	6.15 ± 1.14	7.35 ± 0.88	+1.20	p < 0.001
Overall competence (sum / overall rating)	28.50 ± 3.1	35.65 ± 2.5	+7.15	p < 0.001

Assessment of knowledge acquisition using pre- and post-test scores revealed a highly significant improvement in cognitive performance following the Mini-CEX-integrated posting. The mean pre-test score was 28.50, which increased to 35.65 in the post-test, yielding a mean improvement of 7.15 points. Paired t-test analysis demonstrated a strong statistical significance ( p < 0.001), indicating substantial knowledge gain after the intervention. Pre- and post-test knowledge scores and paired t-test results are presented in Table 2.

**Table 2:** Knowledge test (n = 20)

Measure	Mean ± SD
Pre-test score (mean)	28.50
Post-test score (mean)	35.65
Mean change (post−Pre)	+7.15 points
Paired t test	t(19) ≈ 16.60
p (two-tailed)	p < 0.0001

**Figure 1:**
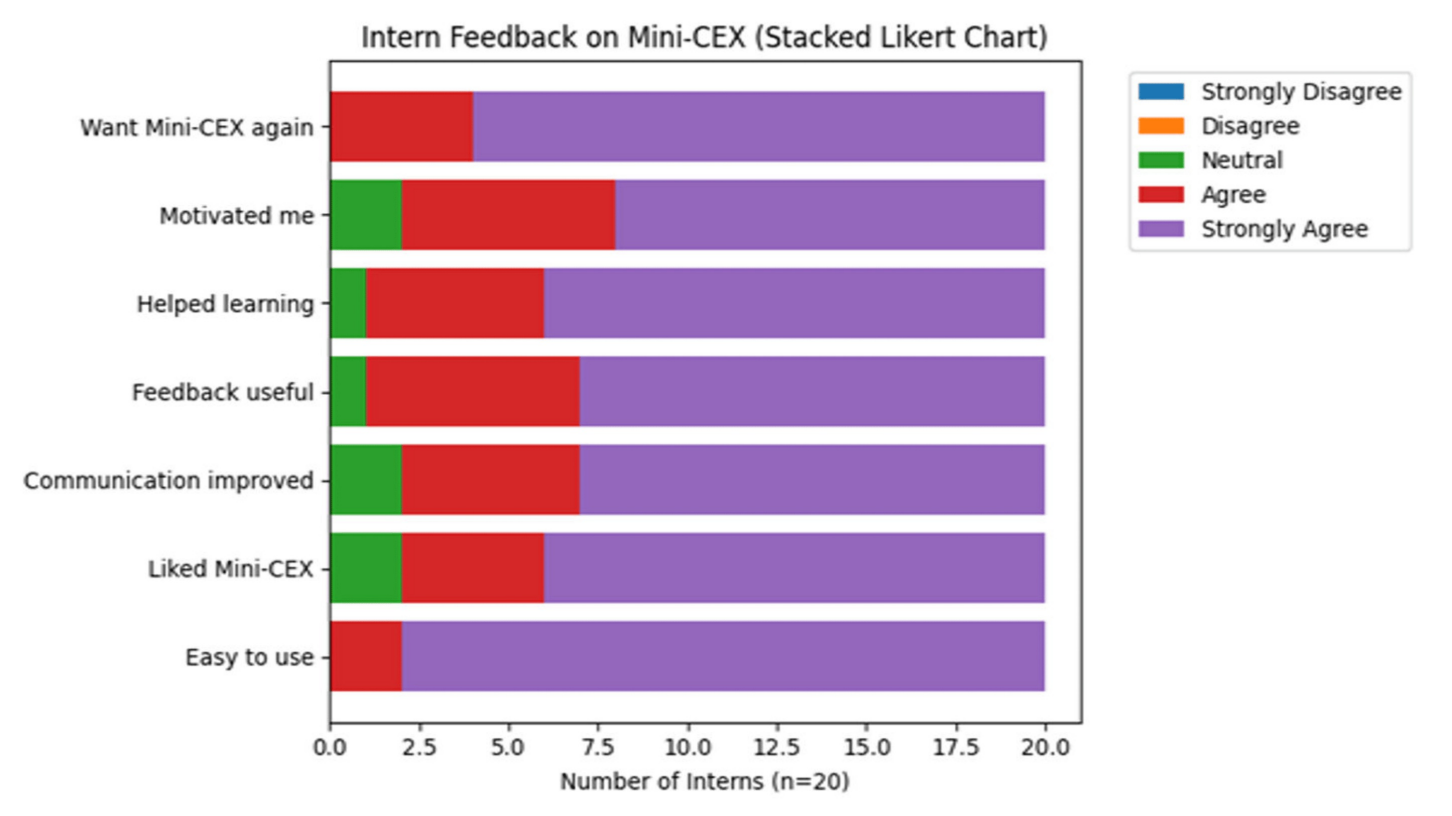
Student feedback

Students' perceptions were analyzed using a stacked Likert scale (n = 20), which demonstrated overwhelmingly positive responses toward Mini-CEX. For all feedback items, 85-95% of interns reported “Agree” or “Strongly Agree.” Mini-CEX was perceived as easy to use by approximately 90% of interns, while nearly 95% felt that it enhanced their learning and improved communication skills. About 85-90% of interns reported increased motivation, found the feedback useful, and felt that Mini-CEX helped them identify their strengths and weaknesses. Importantly, around 90% expressed willingness to undergo Mini-CEX again in future postings, indicating high acceptability of the tool. Intern perceptions of Mini-CEX are illustrated using a stacked Likert scale in Figure 1, showing high levels of agreement across all feedback items.

**Figure 2:**
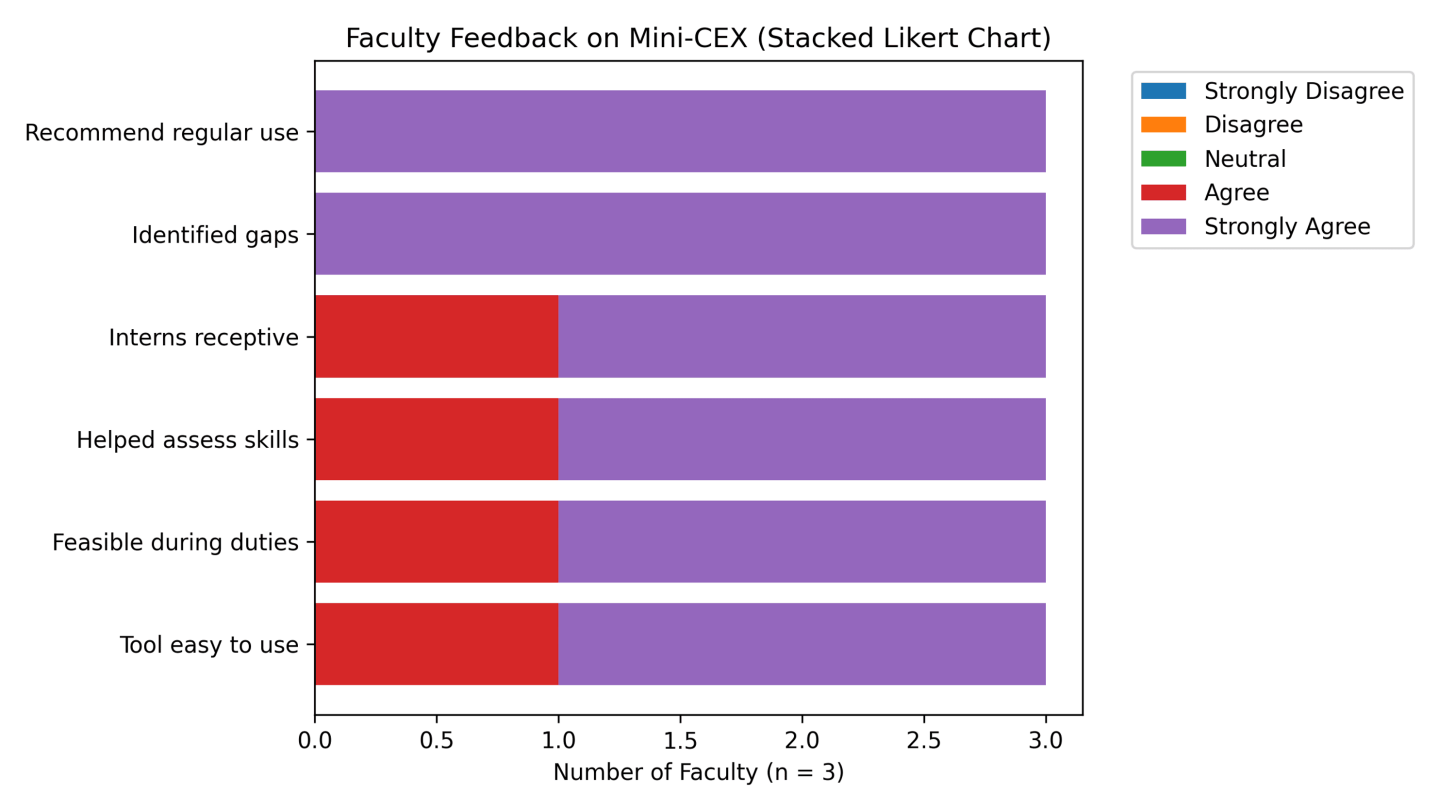
Faculty feedback

Faculty feedback (n = 3) similarly reflected strong acceptance of Mini-CEX as a workplace-based assessment method. All faculty members (100%) agreed or strongly agreed that Mini-CEX was helpful in assessing real-world clinical skills, identifying competency gaps, and recommending its routine use. Faculty perceptions regarding the feasibility and acceptability of Mini-CEX are depicted in Figure 2.

## Discussion

The findings demonstrate that Mini-CEX is a feasible, reliable, and educationally impactful assessment method when implemented even over a short clinical posting. The present study showed a statistically significant improvement across all five Mini-CEX domains-history taking, physical examination, clinical judgment/diagnosis, communication skills, and professionalism-between the first and second encounters. The largest gains were observed in clinical judgment/diagnosis and physical examination, suggesting that direct observation coupled with immediate feedback enhances interns’ diagnostic reasoning and examination skills in respiratory medicine. These findings are consistent with the foundational work by Norcini et al [6], who demonstrated that repeated Mini-CEX encounters lead to measurable improvements in clinical performance across domains, particularly when feedback is structured and timely.

Interns demonstrated a highly significant improvement in knowledge scores following the Mini-CEX-integrated posting (mean increase of 7.15 points; p < 0.001). This supports the idea that workplace assessments can reinforce cognitive learning, likely by prompting focused preparation, reflection, and targeted feedback. Similar findings have been reported by George et al [5] and Nair et al [4], where Mini-CEX-based teaching resulted in improved post-test scores and better retention of clinical concepts.

In the present study, the mean time required per Mini-CEX session was approximately 12-13 minutes, with a 100% completion rate and no reported operational difficulties. This duration is consistent with the brief, focused nature of workplace-based assessments recommended for effective observation and feedback in clinical settings [7,8]. Both interns and faculty reported high levels of satisfaction and acceptance of Mini-CEX. Interns perceived Mini-CEX as helpful in identifying strengths and weaknesses, improving communication skills, and enhancing overall confidence in clinical practice. Faculty members viewed Mini-CEX as a valid and practical tool for assessing real-world clinical competence and identifying learning gaps. These perceptions mirror findings from multiple studies showing that Mini-CEX is well accepted by learners and assessors, especially when used formatively and accompanied by constructive feedback [9]. The quality of Mini-CEX implementation and participant engagement plays an important role in its educational effectiveness [10]. As workplace-based assessment methods are increasingly adopted, there is a growing need for structured faculty development programs in medical colleges to strengthen their implementation and generate further evidence to support their routine use [11].

Limitations

Though we find high acceptability and feasibility, there are a few limitations to this study. It is a single-center, single specialty, single specility and short-duration study. Only two encounters were done per student. Using more encounters, more numbers of interns and using longitudinal follow-up will be more helpful.

## Conclusions

Mini-CEX significantly improves clinical performance across all core domains of respiratory disease diagnosis when used even over a short clinical posting. Knowledge acquisition is enhanced, with a large and statistically significant improvement in post-test scores. Highly acceptable to interns and faculty, promoting engagement, motivation, and reflective learning. Incorporating Mini-CEX routinely during internship can bridge the gap between knowledge and clinical competence. It can be applied to various other clinical departments like internal medicine, surgery, psychiatry and ophthalmology. This will help improve clinical skills in medical interns in all rotating departments.

## Appendices

1. Domain assessment form

A. Domains Assessed (Rated 1-9):

Domain

Rating (1-9)

Comments

History taking

Physical Examination

Clinical Judgment / Diagnosis

Communication Skills

Professionalism

Organization / Time Management

Overall Clinical Competence

B. Feedback Section:

·         Strengths observed: ___________________________

·         Areas for improvement: _________________________

·         Suggestions for learning: _______________________

C. Signatures:

·         Observer/Faculty

·         Intern (to confirm feedback received)

2.

Intern Feedback Form (After Mini-CEX Completion)

Intern Name: ____________________  Date: _______________ Topic:

Feedback statement

Tick one option per row (Strongly Agree = 5, strongly Disagree = 1)

Statement

1<br>Strongly Disagree

2

3

4

5<br>Strongly Agree

The Mini-CEX was easy to understand and participate in

Like to be assessed in my PG residency.

☐

☐

☐

☐

☐

My communication skills improved.

☐

☐

☐

☐

☐

The feedback I received was constructive and useful.

☐

☐

☐

☐

☐

The process helped me identify areas where I need improvement.

☐

☐

☐

☐

☐

Mini-CEX motivated me to prepare better and learn more.

☐

☐

☐

☐

☐

I would like Mini-CEX to be used in other postings, too.

☐

☐

☐

☐

☐

Mention the good things you like about the Mini-CEX session.

Mention things that you disliked about the Mini-CEX session.

Give suggestions to improve the conduct of the Mini-CEX session.

3.

Title: Faculty Name: ____________________  Department: Respiratory Medicine Assessment Method Used: ☐ Mini-CEX

Please rate this on a 5-point scale.

Feedback statement:

(1 = Strongly Disagree, 5 = Strongly Agree)

Statement

1<>Strongly Disagree

2

3

4

5<>Strongly Agree

The Mini-CEX tool was clear and easy to use.

☐

☐

☐

☐

☐

It was feasible to conduct Mini-CEX during clinical duties.

☐

☐

☐

☐

☐

The tool helped me assess interns’ actual clinical skills.

☐

☐

☐

☐

☐

Interns were receptive to the feedback provided.

☐

☐

☐

☐

☐

Able to identify gaps in the knowledge of students.

☐

☐

☐

☐

☐

I would recommend regular use of Mini-CEX for clinical teaching.

☐

☐

☐

☐

☐

Mention good things you like about the Mini-CEX session.

Mention things that you disliked about the Mini-CEX session.

Give suggestions to improve the conduct of the Mini-CEX session.

## References

[1] India State-Level Disease Burden Initiative CRD Collaborators (2018). The burden of chronic respiratory diseases and their heterogeneity across the states of India: The Global Burden of Disease Study 1990-2016. Lancet Glob Health.

[2] Frank JR, Snell LS, Cate OT (2010). Competency-based medical education: Theory to practice. Med Teach.

[3] Kogan JR, Holmboe ES, Hauer KE (2009). Tools for direct observation and assessment of clinical skills of medical trainees: A systematic review. JAMA.

[4] Nair BR, Alexander HG, McGrath BP (2008). The mini clinical evaluation exercise (mini-CEX) for assessing clinical performance of international medical graduates. Med J Aust.

[5] Sivaraman G, Lakshmanan J, Alexander A (2024). Use of mini-Cex as formative assessment tool in the training of undergraduate medical students in ENT situation analysis and the way forward. Indian J Otolaryngol Head Neck Surg.

[6] Norcini JJ, Blank LL, Duffy FD, Fortna GS (2003). The mini-CEX: a method for assessing clinical skills. Ann Intern Med.

[7] Epstein RM (2007). Assessment in medical education. N Engl J Med.

[8] Pelgrim EA, Kramer AW, Mokkink HG, van der Vleuten CP (2012). The process of feedback in workplace-based assessment: Organisation, delivery, continuity. Med Educ.

[9] Khalid H, Chaudhary N, Asif N, Mahmood A, Kamal Z (2024). Mini-CEX as a formative assessment tool for undergraduate medical students in ophthalmology. J Pak Med Assoc.

[10] Gupta S, Sharma M, Singh T (2017). The acceptability and feasibility of mini-clinical evaluation exercise as a learning tool for pediatric postgraduate students. Int J Appl Basic Med Res.

[11] Guraya SY (2015). Workplace-based assessment; applications and educational impact. Malays J Med Sci.

